# Real-Time Impact of COVID-19 on Clinical Care and Treatment of Patients with Tuberculosis: A Multicenter Cross-Sectional Study in Addis Ababa, Ethiopia

**DOI:** 10.5334/aogh.3481

**Published:** 2021-11-15

**Authors:** Dagmawi Chilot, Yimtubezinash Woldeamanuel, Tsegahun Manyazewal

**Affiliations:** 1 Addis Ababa University, College of Health Sciences, Center for Innovative Drug Development and Therapeutic Trials for Africa (CDT-Africa), Addis Ababa, Ethiopia; 2 Department of Physiology, College of Medicine and Health Sciences, University of Gondar, Gondar, Ethiopia

## Abstract

**Background:**

There were global concerns and predictions that Coronavirus disease 2019 (COVID-19) would severely affect tuberculosis (TB) care and treatment services in resource-constrained countries. This study aimed to assess the real-time impact of COVID-19 on clinical care and treatment of patients with TB in Addis Ababa, Ethiopia.

**Methods:**

This was a facility-based, multicenter, cross-sectional study conducted in 10 health centers with high TB clients in Addis Ababa, Ethiopia. Participants were patients with TB who have been attending TB clinical care and treatment in the COVID-19 pandemic period. Data were collected using adapted, interviewer-administered questionnaires to investigate the impact of COVID-19 in their routine care and treatment.

**Result:**

The study included a total of 212 consented participants. Study participants who missed appointments for medication refill were 40 (18.9%). The most important predictors of missed appointments were fear of COVID-19 [AOR = 4.25, 95% CI (1.710–25.446)], transport disruption [AOR = 8.88, 95% CI (1.618–48.761)], lockdown [AOR = 6.56, 95% CI (1.300–33.131)], traveling costs [AOR = 10.26, 95% CI (1.552–67.882)], and personal protective equipment costs [AOR = 11.15, 95% CI (2.164–57.437)]. The most costly COVID-19 preventive measures that caused financial burden to the patients were face mask [107 (50.5%)], disinfectant [106 (50%)], and sop [50 (23.6%)]. The participants were well aware of the recommended COVID-19 preventive measures. Their perceived most effective preventive measures were the use of face mask (90.1%), frequent hand washing with soap and use of disinfectant (83.0%), avoid touching eyes, nose and mouth with unwashed hands (77.8%), and stay at home (75.5%).

**Conclusions:**

COVID-19 significantly hampered the clinical care and treatment of patients with TB. The impact was primarily on their appointments for scheduled medication refills, clinical follow-ups, and laboratory follow-ups. Fear of getting infected with COVID-19, limited access to transportation, reduced income for traveling to health facilities, costs for personal protective equipment and traveling to healthcare facilities, and the lockdown were the major determinants. The impact could be mitigated by reducing the number of visits, rationing personal protective equipment as feasible, compensating travel expenses, providing health educations and community-based TB services, and maintaining TB services.

## Background

The Coronavirus disease 2019 (COVID-19) outbreak has caused severe disruptions to healthcare systems and the overall well-being of people around the world [1,2,3]. There is a global concern that the world’s robust response and investment against the pandemic would impact the already overburdened healthcare system [4,5,6,7]. In Ethiopia, a country in sub-Saharan Africa, the burden of infectious diseases, coupled with shortage of healthcare facilities, healthcare workforce, and personal protective equipment could trigger the spread of the pandemic in the country [8,9,10,11,12,13,14,15]. The country confirmed its first case of COVID-19 on March 13, 2020, and as of June 11, 2021, there have been 273 678 people confirmed positive, of whom 4 231 died (case fatality rate = 1.55%) and 249 028 (90.99%) recovered [16]. Ethiopia has documented success in reducing common infectious diseases in the last two-and-a-half decades, while diseases such as tuberculosis (TB), HIV, lower respiratory infections, and diarrheal diseases are still the major causes of morbidity and mortality in the country [17,18,19,20,21,22,23].

TB is still one of the top 10 causes of death globally. According to the WHO global TB report 2020 [24], an estimated 10 million people fell ill with TB annually. In Ethiopia, although there is a major decline in TB-associated death and incidence, the country is still among the 30 high TB, TB/HIV, and MDR-TB burden countries [24]. The Ethiopian government has been taking key measures to fight TB, and as a result, Ethiopia is among the seven high-burden countries that reached the first milestone of the End TB Strategy: a 20% reduction of TB incidence between 2015 and 2020. However, facilitated by co-infections like HIV/AIDS, and socioeconomic situations including poverty and inequality, TB remains a major challenge in Ethiopia.

Studies conducted in different countries indicated a potential bidirectional link between TB and COVID-19. Patients with TB are more likely to experience poor outcomes from COVID-19 [25] and patients co-infected with COVID-19 and TB are more likely to suffer severe disease or death than patients with COVID-19 only [26]. The simultaneity of COVID-19 and pulmonary TB can issue a diagnostic dilemma [27] and new diagnostic challenges for clinicians [28]. As both TB and COVID-19 share respiratory symptoms, similar infrastructure and expertise are needed for the diagnosis, management and containment of both diseases [29,30]. COVID-19 creates stress among TB patients to go to healthcare facilities for diagnosis and treatment and the pandemic’s extensive demand for healthcare providers challenges the provision of routine TB case follow-ups [31,32,33]. As a result, different countries have attempted to adjust their TB care and treatment procedures based on their local demands and contexts, including multi-month dispensing and postal delivery of TB medications, home supply of TB treatments, outreach services to reach TB patients, and the use of digital health technologies to monitor medication intakes [34,35,36,37]. However, patient-level studies have been limited to inform how effective and useful these interventions were for the patients.

In Ethiopia, there have been limited studies conducted in the area, reporting that the series of COVID-19 containment measures that the government had taken, including state of emergency, had a significant impact on the overall TB care and treatment services in the country including a reduction in the flow of TB patients [38,39]. However, there has been no study that documented the impact of COVID-19 on patients with TB using real-time patient-level data.

Thus, this study aimed to assess the real-time impact of COVID-19 on the clinical care and treatment of patients with TB in Addis Ababa, Ethiopia.

## Methods

### Design and setting

A health facility-based, multicenter, cross-sectional study was conducted in ten primary healthcare facilities in Addis Ababa, Ethiopia, from January 15 to February 30, 2021. According to United Nations population estimates, Addis Ababa’s population is estimated at 4 793 699 in 2020. The city is administratively divided into ten sub-cities and 116 districts. Addis Ababa Health Bureau is responsible for the overall health activity in the city. There are numerous healthcare facilities in the city, including six public hospitals and 106 public health centers in the city. COVID-19 pandemic and its transmission potential has been relatively higher in Addis Ababa [39]. As a lot of people live in the city under crowded housing conditions, the risk for TB transmission is high in the city [18].

The public health centers were stratified by sub-city and one site with high TB patient’s load was taken from each sub-city, with a total of 10 facilities included. The study facilities were Addis Raey Health Center (Addisketema), Akaki Health Center (Akaki kality), Kebena Health Center (Arada), Goro Health Center (Bole), Adisu Gebeya Health Center (Gulele), Kazanchis Health Center (Kirkos), Alem Bank Health Center (Kolfe), Teklehaymanot Health Center (Lideta), Woreda 02 Health Center (Nifasilk lafto), and Woreda 13 Health Center (Yeka).

### Participants

**Figure 1:**
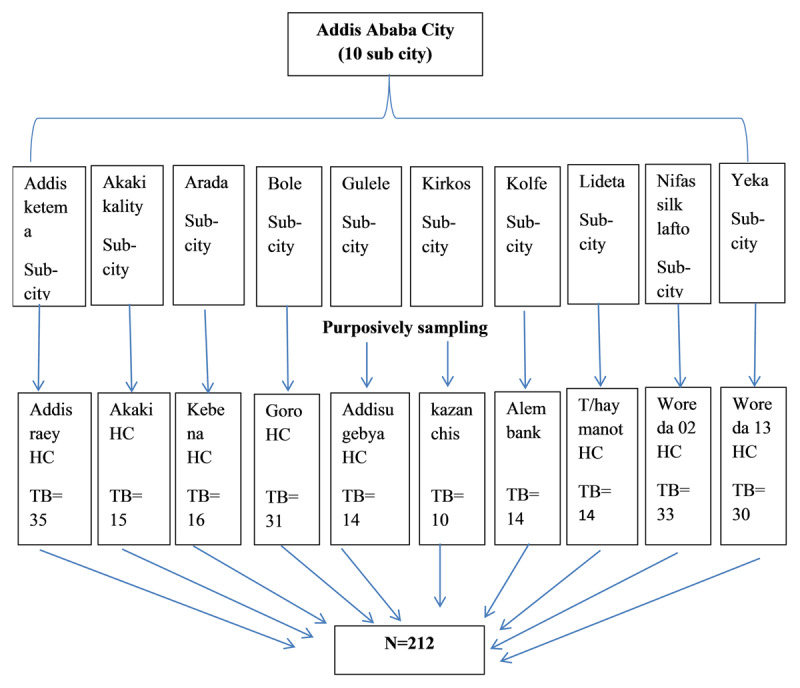
Schematic presentation of sampling procedure.

The source population was all TB outpatients of age >18 years attending care and treatment in the selected health facilities, and the study population was patients who had a confirmed diagnosis of any types of TB, regardless of whether the TB is drug-susceptible or drug-resistant, and attending TB clinical care and treatment in the study facilities during the data collection period. Thus, participants were included if they were 1) patients with TB, as confirmed within the study facilities or result referred from another health facility, attending care and treatment services in the study facilities during the data collection period; 2) man or woman aged ≥ 18 years; 3) volunteers to participate in the study. All participants who have been attending their TB clinical care and treatment in the study sites during the data collection period were considered using a predetermined sampling procedure (***Figure 1***).

### Data collection

We used an adapted, pre-tested, close-ended, interviewer-administered questionnaire to collect primary data from the study participants for the assessment of the overall impact of COVID-19. It had sections relevant to socio-demographic characteristics, awareness on COVID-19, patient-level preventive measures, patient-level financial burdens, TB care and treatment services, barriers to access healthcare, precaution measures in the healthcare facilities, medication and follow-ups, and non-medical supports during the COVID-19 period. The questionnaire was developed in English and translated to the local national language (Amharic), and later be back-translated to English. When they came to their routine TB care services, the participants were given information about the study through an information sheet and to sign a consent form if they agree and involve in the study. Ten data collectors and two supervisors were recruited and trained for a half-day on data collection techniques and involved accordingly.

### Data analysis and interpretation

The data collection tool was checked for completeness and internal consistency. Then, data entry and cleaning were done by the principal investigator. The analysis was done using bivariate and multivariate logistic regression to observe the effects of independent variables on the outcome variable while simultaneously controlling for other potential confounding factors. The raw data was entered to Epi Info version 7 and exported to SPSS 26 for analysis.

## Results

### Socio-demographic characteristics

A total of 212 TB patients were enrolled in the study, with a response rate of 100%, and 110 (51.9%) were female. Of the total, 97 (45.8%) and 64 (30.2%) were in the age group 35–54 and 18–34 years, respectively. Ninty-five (44.8%) were married and 57 (26.9%) had attended primary education. One hundred and twenty-seven (59.9%) were Orthodox Christian, and fifty-two (24.5%) were unemployed (***Table 1***).

**Table 1:** Sociodemographic characteristics of participants, Addis Ababa, Ethiopia.


VARIABLE	CATEGORY	FREQUENCY	%

Sex	Male	102	48.1%

	Female	110	51.9%

Health center name	Addis raey	35	16.5%

	Akaki	15	7.1%

	Kebena	16	7.5%

	Goro	31	14.6%

	Addisu gebya	14	6.6%

	Kazanchis	10	4.7%

	Alembank	14	6.6%

	T/haymanot	14	6.6%

	Woreda 02	33	15.6%

	Woreda 13	30	14.2%

Age	18–34	64	30.2%

	35–54	97	45.8%

	≥55	51	24.1%

Marital status	Single	59	27.8%

	Married	95	44.8%

	Widowed	25	11.8%

	Divorced	21	9.9%

	Separated	12	5.7%

Level of education	No formal education, cannot read or write	28	13.2%

	No formal education, can read or write	41	19.3%

	Primary education	57	26.9%

	Secondary education	44	20.8%

	Diploma and above	42	19.8%

Religion	Orthodox	127	59.9%

	Muslim	51	24.1%

	Protestant	21	9.9%

	Catholic	8	3.8%

	Others	5	2.4%

Occupation	Student	13	6.1%

	Daily laborer	42	19.8%

	Merchant	38	17.9%

	Governmental employee	36	17.0%

	Private/NGO employee	29	13.7%

	Farmer	2	0.9%

	Unemployed	52	24.5%

Family size	One	46	21.7%

	Two	54	25.5%

	≥ three	112	52.8%

Average monthly income	≤1000 Eth. Birr	16	7.5%

	1001–3000 Eth. Birr	49	23.1%

	3001–10000 Eth. Birr	112	52.8%

	>10000 Eth. Birr	35	16.5%


NB: NGO- Non-governmental organization; Eth. Birr-Ethiopian Birr.

### Most effective preventive measure of COVID-19

Of the total study participants, 160 (75.5%) agreed “stay at home” is the most effective preventive measure of COVID-19. According to the participants, the most effective COVID-19 preventive measures were the use of facemask (90.1%), frequent hand washing with soap and use disinfectant (83.0%), avoid touching eyes, nose and mouth with unwashed hands (77.8%), and stay at home (75.5%) (***Table 2***).

**Table 2:** Respondents’ awareness on COVID-19 preventive measures, Addis Ababa, Ethiopia.


VARIABLE	CATEGORY	FREQUENCY	%

Stay at home	Disagree	52	24.5%

	Agree	160	75.5%

Maintain physical distancing	Disagree	72	34.0%

	Agree	140	66.0%

Avoid close contact	Disagree	70	33.0%

	Agree	142	67.0%

Cover mouth and nose with a facemask	Disagree	21	9.9%

	Agree	191	90.1%

Frequent handwashing with soap	Disagree	36	17.0%

	Agree	176	83.0%

Avoid touching of eyes, nose and mouth	Disagree	47	22.2%

	Agree	165	77.8%

Avoid mass gathering	Disagree	78	36.8%

	Agree	134	63.2%

Restrict movement	Disagree	88	41.5%

	Agree	124	58.5%

Use disinfectants	Disagree	36	17.0%

	Agree	176	83.0%


### Financial burden of COVID-19

The most costly COVID-19 preventive measures that cause financial burden to the patients were costs for buying facemasks [107 (50.5%)], sops for handwashing [50 (23.6%)], and disinfectants [106 (50.0%)] (***Table 3***).

**Table 3:** Participants’ financial burden of COVID-19 preventive measures, Addis Ababa, Ethiopia.


VARIABLE	CATEGORY	FREQUENCY	%

Stay at home	No	212	100.0%

	Yes	0	0.0%

Cover mouth and nose with facemask	No	105	49.5%

	Yes	107	50.5%

Wash hands with soap frequently	No	162	76.4%

	Yes	50	23.6%

Use disinfectants as asppropriate	No	106	50.0%

	Yes	106	50.0%


### TB care and treatment services during COVID-19

All of the participants have gone to a healthcare facility during the high COVID-19 time. Of these, 173 (81.6%) were treated differently. One hundred and thirty-one (61.8%) passed through new procedures, and for most participants, the procedure was a COVID-19 PCR test (52.7%). Six (2.8%) participants were obliged to change the health facility, and 33 (15.6%) have ever been denied health services because of this pandemic. Almost all participants said healthcare providers are polite and respectful (99.5%), willing to listen and answer their questions (99.5%), give attention to their individual needs (98.1%), and never physically assaulted (99.5%) (***Table 4***).

**Table 4:** Participants’ response on health care facility and service delivery during COVID-19, Addis Ababa, Ethiopia.


VARIABLE	CATEGORY	FREQUENCY	%

Visits the facility during high COVID-19 time	No	0	0.0%

	Yes	212	100.0%

Treated differently	Yes	173	81.6%

	No	39	18.4%

Unusual procedures were in place	Yes	131	75.7%

	No	42	24.3%

Unusual procedure (if any)	COVID-19 creening	62	47.3%

	COVID-19 PCR	69	52.7%

	COVID-19 chest CT	0	0.0%

	Others	0	0.0%

Obliged to change the health center because of COVID-19	Yes	6	2.8%

	No	206	97.2%

Denied healthcare services	Yes	33	15.6%

	No	179	84.4%

Obtained polite and respectful services	Yes	211	99.5%

	No	1	0.5%

Listened well and got satisfactory answers to queries	Yes	211	99.5%

	No	1	0.5%

Gained considerable attention regarding individual needs	Yes	208	98.1%

	No	4	1.9%

Physically assaulted by providers	Yes	1	0.5%

	No	211	99.5%

Received prompt action for your health conditions or comorbidities	Yes	128	60.4%

	No	84	49.6%


*NB*: PCR- polymerase chain reaction; CT- computed tomography; COVID-19- coronavirus disease 2019.

### COVID-19 precaution measures in healthcare facilities

From the total study participants, 208 (98.1%) responded that health professionals provide health education on COVID-19, and 133 (62.7%) said the health centers provide COVID-19 screening. According to the participants, almost all health professionals wear gloves (99.5%) and mask (99.5%) when providing care, and there was frequent access to water (95.8%) and soap (95.3%) at the gate of the healthcare facilities, but not sanitizer (62.3%) (***Table 5***).

**Table 5:** Response of study participants on health care facility and service delivery during COVID-19, Addis Ababa, Ethiopia.


VARIABLE	CATEGORY	FREQUENCY	%

Providers give health education on COVID-19	Agree	208	98.1%

	Disagree	4	1.9%

Health center provides COVID-19 screening	Agree	133	62.7%

	Disagree	79	37.3%

Providers wear gloves during caregiving	Agree	211	99.5%

	Disagree	1	0.5%

Providers wear mask during caregiving	Agree	211	99.5%

	Disagree	1	0.5%

Health center has water at entry for handwashing	Agree	203	95.8%

	Disagree	9	4.2%

Health center has soap at entry for handwashing	Agree	202	95.3%

	Disagree	10	4.7%

Health center has sanitizer at entry for hand cleaning	Agree	80	37.7%

	Disagree	132	62.3%


### Medications and follow-ups during COVID-19

In this study, 199 (93.9%) of the participants responded the pharmacy was accessible, and all participants had taken their drugs. Participants who missed appointments, follow-up tests, and counseling services were 40 (18.9%), 41 (19.3%), and 37 (17.5%) respectively according to their response (***Table 6***).

**Table 6:** Response of study participants on medications and follow-up during COVID-19, Addis Ababa, Ethiopia.


VARIABLE	CATEGORY	FREQUENCY	%

Accessed pharmacy readily	Yes	199	93.9%

	No	13	6.1%

Ordered with drugs and supplies	Yes	212	100.0%

	No	0	0.0%

Missed appointments or visits	Yes	40	18.9%

	No	172	81.1%

Obtained follow-up laboratory tests	Yes	171	80.7%

	No	41	19.3%

Obtained follow-up counseling on medication or health status	Yes	175	82.5%

	No	37	17.5%


### Missing appointments/visits for medication refill

On bivariate and multivariate logistic regression analysis, the independent variables marital status, education, monthly income, fear of COVID- 19, transport disruption, reduced income, immediate access to provider, soap available, pharmacy accessible, shortage of medicine and non-medical support since COVID-19 had significant associations with missing appointments/visits for medication refill during the COVID-19 pandemic (***Tables 7, 8, 9***).

**Table 7:** Bivariate and multivariate logistic regression analysis of appointments for refill during COVID-19, Addis Ababa, Ethiopia.


		MISSED APPOINTMENTS		ODDS RATIO		

VARIABLES	CATEGORY	NO	YES	COR (CI)	AOR (CI)	P VALUE

Fear of COVID-19	No	45 (26.2%)	1 (2.5%)	1	1	

	Yes	127 (73.8%)	39 (97.5%)	40.93 (13.575–123.437)	4.25 (1.710–25.446)	0.013*

Transport disruption	No	87 (50.6%)	4 (10.0%)	1	1	

	Yes	85 (49.4%)	36 (90.0%)	9.21 (3.143–27.000)	8.88 (1.618–48.761)	0.012*

Partial lockdown	No	68 (39.5%)	1 (2.5%)	1	1	

	Yes	104 (60.5%)	39 (97.5%)	47.73 (16.896–134.815)	6.56 (1.300–33.131)	0.023*

Reduced income to travel	No	53 (30.8%)	1 (2.5%)	1	1	

	Yes	119 (69.2%)	39 (97.5%)	50.53 (11.725–217.789)	10.26 (1.552–67.882)	0.016*

Unable to access mask	No	81 (47.1%)	2 (5.0%)	1	1	

	Yes	91 (52.9%)	38 (95.0%)	145.80 (0.283–491.13)	11.15 (2.164–57.437)	0.004*

Staff seem uncomfortable	No	120 (69.8%)	21 (52.5%)	1	1	

	Yes	52 (30.2%)	19 (47.5%)	36.00 (12.972–99.909)	5.69 (1.174–27.602)	0.031*


**Table 8:** Bivariate and multivariate logistic regression analysis of follow-up tests during COVID-19, Addis Ababa, Ethiopia.


		FOLLOW-UP TEST		ODDS RATIO		

VARIABLES	CATEGORY	NO	YES	COR (CI)	AOR (CI)	P VALUE

Age	18–34	7 (17.1%)	57 (33.3%)	1	1	

	35–54	8 (19.5%)	89 (52.1%)	1.56 (0.433–5.635)	3.28 (0.483–22.327)	0.224

	≥55	26 (63.4%)	25 (14.6%)	0.07 (0.025–0.203)	0.14 (0.022–0.836)	0.031*

Partial	No	1 (2.4%)	68 (39.8%)	1	1	

Lockdown	Yes	40 (97.6%)	103 (60.2%)	0.03 (0.010–0.067)	0.16 (0.028–0.903)	0.038*

Transport disruption	No	5 (12.2%)	86 (50.3%)	1	1	

	Yes	36 (87.8%)	85 (49.7%)	0.14 (0.051–0.367)	0.18 (0.043–0.800)	0.024*

Fear of COVID-19	No	2 (4.9%)	44 (25.7%)	1	1	

	Yes	39 (95.1%)	127 (74.3%)	0.03 (0.011–0.085)	0.12 (0.019–0.779)	0.026*

Unable to access mask	No	3 (7.3%)	80 (46.8%)	1	1	

	Yes	38 (92.7%)	91 (53.2%)	0.01 (0.003–0.027	0.06 (0.009–0.374)	0.003*

Denied health services	No	15 (36.6%)	159 (93.0%)	1	1	

	Yes	26 (63.4%)	12 (7.0%)	0.04 (0.018–0.103)	0.11 (0.020–0.641)	0.014*


* Statistically significant at p-value < 0.05, COR = crude odds ratio at 95% confidence interval; AOR = adjusted odds ratio at 95% confidence interval.

**Table 9:** Bivariate and multivariate logistic regression analysis of counseling during the COVID-19 pandemic, Addis Ababa, Ethiopia.


		COUNSELING DONE		ODDS RATIO		

VARIABLE	CATEGORY	NO	YES	COR (CI)	AOR (CI)	P VALUE

Age	18–34	3 (8.1%)	59 (33.7%)	1	1	

	35–54	5 (13.5%)	89 (50.9%)	0.90 (0.208–3.931)	0.33 (0.022–4.805)	0.414

	≥55	29 (78.4%)	27 (15.4%)	0.05 (0.013–0.169)	0.06 (0.005–0.905)	0.042*

Partial lockdown	No	1 (2.7%)	68 (38.9%)	1	1	

	Yes	36 (97.3%)	107 (61.1%)	0.03 (0.009–0.073)	0.03 (0.001–0.772)	0.034*

Fear of COVID-19	No	1 (2.7%)	45 (25.7%)	1	1	

	Yes	36 (97.3%)	130 (74.3%)	0.02 (0.006–0.071)	0.03 (0.002–0.532)	0.017*

Stigma	No	14 (37.8%)	173 (98.9%)	1	1	

	Yes	23 (62.2%)	2 (1.1%)	0.01 (0.002–0.033)	0.01 (0.000–0.115)	0.002*


* Statistically significant at p-value < 0.05, COR = crude odds ratio at 95% confidence interval; AOR = adjusted odds ratio at 95% confidence interval.

### Discussion

This study assessed the real-time, patient-level impact of COVID-19 on the clinical care and treatment of patients with TB in Addis Ababa, Ethiopia. With a total of 212 patients with TB included, 18.9% of the participants missed appointments for medication refill in the COVID-19 outbreak for reasons including fear of COVID-19 infection, transport disruption, partial lockdown, reduced income for traveling to a healthcare facility, unable to get face masks, and pressure placed on providers. The participants were well aware of the COVID-19 preventive measures, where the most effective COVID-19 preventive measures they considered were the use of facemask, frequent hand washing with soap and use disinfectant, avoid touching eyes, nose and mouth with unwashed hands, and stay at home. The most costly COVID-19 preventive measures that caused a financial burden to the patients were facemask, disinfectant, and sop. To the best of our knowledge, this study was the first of its kind to assess the impact of COVID-19 on TB care and treatment services in Ethiopia using real-time, patient-level data.

The analysis indicated that fear of getting COVID-19 infection within the healthcare facilities a strong predictor of missing medication appointments. TB treatment disruption could abate patient outcomes and lead to TB drug resistance. In many sub-Saharan African countries, the COVID-19 pandemic has added burdens to already overwhelmed health systems and the burden of TB may aggravate as a result [40]. Applying a combination of standard infection control measures, healthcare facilities need to improve patients’ trust and keep them safe from the pandemic.

Transport disruption, particularly in the partial lockdown period, was among the main barriers for the TB patients, limiting their access to healthcare facilities thereby missing follow-up visits. The finding was in agreement with previous studies conducted in Ethiopia [11,39,41,42,43] and elsewhere in Africa [44,45,46,47,48] that COVID-19 lockdowns had a significant impact on patients’ access to transportation to healthcare facilities. In the case of TB medication follow-up visits, direct-observed therapy procedure necessitates patients’ daily visits to healthcare facilities for medication intake this procedure is susceptible to transport disruptions [6,39,40]. An appropriate people-centered model of care needs to be developed in consultation with TB patients and TB survivors to mitigate the challenges and upsurge the TB services [49]. To minimize the challenge, many countries, including Ethiopia, had modified their TB care service delivery by reducing patients’ health facility visits during the COVID-19. More advanced COVID-19 mitigation strategies, multilateral collaborations, international joint-venture, and innovative technologies are essential to address the double burden of the COVID-19 pandemic [50,51,52,53].

In the early days of the COVID-19 pandemic, there was limited access to facemasks for public use in Ethiopia. This was reverted shortly through huge donor and commercial supply and local production. However, our analysis demonstrates that despite understanding the protective effect of masks, patients with TB could not afford to buy facemask. This may be mitigated by rationing of masks for the patients until they complete their medications successfully.

There were some limitations to this study. The study was limited to healthcare facilities in Addis Ababa, and therefore may not be representative of Ethiopia. As the study design was a cross-sectional study, it does not show a causal relationship and only provides a view of the impacts of COVID-19 in a specific period. Otherwise, the study was based on real-time, patient-level primary data and it was conducted in a resource-constrained, high TB burden country context.

## Conclusion

COVID-19 significantly hampered the clinical care and treatment of patients with TB. The impact was primarily on their appointments for scheduled medication refills, clinical follow-ups, and laboratory follow-ups. Fear of getting infected with COVID-19, limited access to transportation, reduced income for traveling to health facilities, costs for personal protective equipment and traveling to healthcare facilities, and the lockdown were the major determinants. The impact could be mitigated by reducing the number of visits, rationing personal protective equipment as feasible, compensating travel expenses, providing health educations and community-based TB services, and maintaining TB services.

## Abbreviations

AAU, Addis Ababa University; COVID-19, Coronavirus Disease 2019; SARS-CoV-2, Severe acute respiratory syndrome coronavirus 2; TB, Tuberculosis; DOT, Directly observed therapy; HC, Health center; ICU, Intensive care unit; MDR, Multidrug-resistant tuberculosis; PPE, Personal protective equipment; WHO, World Health Organization; AOR, Adjusted Odds Ratio; CI, Confidence Interval.
